# Evaluation of Seaweed Extracts From *Laminaria* and *Ascophyllum nodosum* spp. as Biostimulants in *Zea mays* L. Using a Combination of Chemical, Biochemical and Morphological Approaches

**DOI:** 10.3389/fpls.2018.00428

**Published:** 2018-04-06

**Authors:** Andrea Ertani, Ornella Francioso, Anna Tinti, Michela Schiavon, Diego Pizzeghello, Serenella Nardi

**Affiliations:** ^1^Dipartimento di Agronomia, Animali, Alimenti, Risorse Naturali e Ambiente, Università di Padova, Padova, Italy; ^2^Dipartimento di Scienze e Tecnologie Agro-Alimentari, Università di Bologna, Bologna, Italy; ^3^Dipartimento di Scienze Biomediche e Neuromotorie, Università di Bologna, Bologna, Italy

**Keywords:** nutrition, root morphology, spectroscopy, hormones, esterase, maize, phenols

## Abstract

Seaweed extracts can be employed as biostimulants during crop cultivation owing to their positive effects on plant performance. Therefore, in this study one extract from *Laminaria* (A) and five extracts from *Ascophyllum nodosum* (B–F) were assayed on maize (*Zea mays* L.) plants supplied for 2 days with 0.5 mL L^−1^ of single products to evaluate their capacity to stimulate root growth and morphology, nutrition, and sugars accumulation. Firstly, extracts were chemically characterized via Fourier transform infrared (FT-IR) and FT-Raman spectroscopies, and their content in carbon, nitrogen, phenolic acids and hormones (indole-3-acetic acid, IAA, and Isopentenyladenosine, IPA) was quantified. The auxin like- and gibberellic acid -like activities of all extracts were also determined. FT-IR and FT-Raman spectra provided complementary information depicting distinct spectral pattern for each extract. Bands assigned to alginic and uronic acids were dominant in FT-IR spectra, while those corresponding to polyaromatic rings were evident in FT-Raman spectra. In general, extracts stimulated root growth, nutrition, esterase activity, and sugar content. However, they showed high variation in chemical features, which may explain their different capacity in triggering physiological responses in maize. Among *A. nodosum* extracts for instance, E was the most efficient in promoting root morphology traits, likely because of its elevate content in IAA (32.43 nM), while F extract was the highest in phenol content (1,933 mg L^−1^) and the most successful in improving plant nutrition. On the other hand, C extract was very effective in stimulating root elongation, but did not influence plant nutrition. B and D extracts induced similar positive effects on plants, although they greatly varied in chemical composition. *Laminaria* extract (A) differed from *A. nodosum* extracts, because of its low content in total phenols and the presence of both IAA- and GA-like activity. We conclude that all seaweed extracts acted as biostimulants in maize, but their chemical properties appeared crucial in predicting the physiological response preferentially elicited by individual seaweed extracts.

## Introduction

Consumers are increasingly appreciating the production of high quality, healthy fruits and vegetables, especially when they are obtained through a minimal impact on the environment (Spinelli et al., 2010). New approaches have been proposed to grow up the sustainability of agricultural productions and improve the quality of crops and crop-derived products. A promising tool is the use of biostimulants in the form of mixtures of substances and/or microorganisms, which are able to increase the plant nutrient use efficiency and tolerance to abiotic and biotic stresses (Colla et al., 2015; Nardi et al., 2016).

The mechanisms activated by biostimulants in plants are difficult to establish owing to the complex pool of bioactive molecules present in their formulation (Ertani et al., 2011, 2013a, 2014; Guinan et al., 2013). Studying the effects triggered by individual components might produce incorrect results when compared to those determined by the combination, and synergistic action of all constituents in the mixtures may outcome. In addition, the effects of biostimulants are not always consistent among the plant species. This is likely because plants can exhibit different sensitivity thresholds to one or more bioactive molecules (Colla et al., 2015). Also, the extraction method from the source material is critical for the maintenance of the activity of the mixture components, and different extraction procedures from the same matrix may produce more biostimulants with distinct properties and effectiveness (Godlewska et al., 2016; Michalak et al., 2016).

Among biostimulants, a special attention is given to seaweed extracts (Blunden, 1991; Cassan et al., 1992; Calvo et al., 2014). The brown seaweeds *Phaeophyceae* in particular, are employed in sustainable agricultural applications (Goñi et al., 2016), and some of them like *Ascophyllum nodosum, Macrocystis pyrifera*, and *Durvillea potatorum* are widely used in food and industrial applications (Khan et al., 2009). Seaweed extracts elicit an array of positive responses in plants (Prithiviraj, 2008), including the increase in growth, germination rate, chlorophyll synthesis, fruit quality, and post-harvest shelf life (Hong et al., 2007; Khan et al., 2009; Pereira et al., 2009; Calvo et al., 2014; Goñi et al., 2016). They can also induce earlier germination, flowering, and fructification (Mancuso et al., 2006; Sivasankari et al., 2006; Roussos et al., 2009; Ali et al., 2015; Satish et al., 2015), stimulate the proliferation of secondary roots (Mugnai et al., 2008; Pereira et al., 2009; Spinelli et al., 2010) and induce immunity/resistance to pathogens and abiotic stress in plants (Joubert and Lefranc, 2008; Sharma et al., 2014). Recently, the capacity of a commercial extract of *Ascophyllum nodosum* to alleviate drought stress in soybean via changes in physiology and expression of stress-related genes has been reported (Santaniello et al., 2017).

To date, several compounds have been identified in seaweeds as activators of plant defense mechanisms and growth promoters. Among them are: (i) polyphenols, such as phloroglucinol and its derivate eckol (Craigie, 2011; González et al., 2013, 2014; Synytsya et al., 2014; Rengasamy et al., 2015); (ii) polysaccharides, primarily alginate, fucoidan, laminaran, and carrageenans or their derived oligosaccharides (Chandía et al., 2004; Chandía and Matsuhiro, 2008; Khan et al., 2009; Craigie, 2011; González et al., 2014; Rengasamy et al., 2015); (iii) betaines, amino acids, and vitamins (MacKinnon et al., 2010); (iv) substances displaying hormone-like activity. Another valuable active component of seaweed extracts is kahydrin, a derivative of vitamin K1, which favors the secretion of H^+^ ions into the apoplast (Lüthje and Böttger, 1995). This process leads to acidification of the rhizosphere, thus changing the redox state of soil and the availability of nutrients to plants.

Despite the chemical constituents of brown seaweed extracts and the physiological effects they can trigger in plants have been widely described by Battacharyya et al. (2015) with a special emphasis on horticultural crops, the comparison between several extracts with respect to their capacity to induce preferential physiological responses in plants based on the characterization of their chemical composition looks relevant in view of predicting seaweed extract specific biostimulatory effects. Therefore, this research was aimed to characterize the chemical properties and the hormone-like activity of different commercial extracts from *Laminaria* and *Ascophyllum nodosum* spp., and to explore their effects on some aspects of maize plants physiology. The main objective was to find a robust relationship between the specific properties of individual biostimulants and the physiological responses preferentially elicited, which may allow the prediction of metabolic targets for other commercial seaweed biostimulants. The choice of commercial seaweed extracts was dictated by the great variety of products that are available in the market of biostimulants, which may differ based on the algal species, and addition of chemicals or bioactive molecules during the manufacturing process.

## Materials and methods

### Spectroscopic characterization of seaweed extracts

Six commercially available liquid seaweed extracts, one from *Laminaria* spp. and five from *A. nodosum* spp. of North Europe origin, were used in this study. They were produced via extraction with water acidified with sulfuric acid to pH 3–3.5. The mixtures were then centrifuged and the pH adjusted near neutral by addition of potassium hydroxide. Finally, the extracts were sieved through cellulosic membrane filters at 0.8 μm (Membra-Fil® Whatman Brand, Whatman, Milano, Italy). Each extract was classified with different letters: the extract derived from *Laminaria* was named A, while those obtained from *Ascophyllum nodosum* spp. were named from B to F. Extracts were freeze-dried before performing chemical and spectroscopic analyses.

The FT-IR spectra of lyophilized extracts were recorded using an ALPHA FTIR spectrometer (Bruker Optics, Ettlingen, Germany) equipped with an ATR (attenuated total reflectance) sampling device containing diamond crystal. The absorbance spectra were collected between 4,000 cm^−1^ and 400 cm^−1^, at a spectral resolution of 4 cm^−1^, with 64 scans co-added and averaged. A background spectrum of air was scanned under the same instrumental conditions before each series of measurements. Spectra were processed with the Grams/386 spectroscopic software (version 6.00, Galactic Industries Corporation, Salem, NH).

Raman spectra of lyophilized extracts were recorded in solid state with a Multiram FT-Raman spectrometer (Bruker Optics, Ettlingen, Germany) equipped with a cooled Ge-diode detector. The spectral resolution was 4 cm^−1^ and 6,000 scans for each spectrum were collected (integration time about 4 h). The excitation source was a Nd^3+^-YAG laser (1,064 nm, about 30 mW laser power on the sample) in the backscattering (180°) configuration. The low laser power was due to the brown color of the samples which burned out using a higher laser power.

B extract in particular, appeared fluorescent and enriched in mineral content, therefore both its FT-Raman and FT-IR spectra were not comparable to the others (not shown).

### Determination of total carbon and nitrogen, phenols and hormones (IAA and IPA) in seaweed extracts

Carbon (C) and nitrogen (N) contents of seaweed extracts were determined using a dry combustion procedure inside an element analyser (vario MACRO CNS, Hanau, Germany).

Total phenols were quantified via the Folin method according to Arnaldos et al. (2001). Phenols were extracted in water/methanol (1:1 v/v) and filtered at 0.45 μm. The separation of phenols was carried out with HPLC 2,700 (Thermo Finnigan, San Jose, CA, USA) coupled with an 1,806 UV/Vis (Thermo Finnigan, San Jose, CA, USA) detector. The column was a TM-LC 18 (Supelcosil) equipped with pre-column TM-LC 18 (Pelliguard, Supelco). Elution was performed at a flow rate of 1.2 mL min^−1^ using as mobile phase a mixture of water/ n-butanol/ acetic acid (80.5:18:1.5 v/v). The sample injection volume was 20 μL. Detection was performed at 275 nm and the identification of compounds was achieved by comparing their retention time values with those of standards (gallic, protocatetic, vanillic, caffeic, *p*-coumaric, syringic, and p-hydroxybenzoic acids (Sigma-Aldrich). The calibration curve and quantification were performed considering the relationship between peak areas vs. standard concentrations at four concentrations *(n* = 4). A linear fitting with a R squared (*R*^2^) = 0.99 was obtained. All reagents were of analytical grade.

Indole-3-acetic acid (IAA), an auxin, and isopentenyladenosine (IPA), a cytokinin, were determined in extracts using an enzyme-linked immuno-sorbent assay (ELISA) (Phytodetek-IAA and Phytodetek-IPA, respectively, Sigma, St. Louis, MO) as reported in Ertani et al. (2013a,b). The ELISA assay used monoclonal antibodies sensitive in the range between 0.05 and 100 pM. The tracer and standard solutions were prepared following the manufacturer's instructions, and absorbance was read at λ = 405 nm with a Biorad microplate reader (Hercules, CA). Additional details were previously reported by Schiavon et al. (2010).

### Hormone-like activities of seaweed extracts

The IAA-like activity was estimated by measuring the reduction of watercress (*Lepidium sativum* L.) roots after treatment with either IAA or seaweed extracts, while the gibberellin-like (GA-like) activity was determined by measuring the increase in the epicotyls length of lettuce (*Lactuca sativa* L.) after GA and seaweed extracts application (Audus, 1972). In detail, watercress and lettuce seeds were surface-sterilized by immersion in 8% hydrogen peroxide for 15 min. After rinsing five times with sterile distilled water, 10 seeds were placed on a sterile filter paper in a sterile Petri dish. For watercress, the filter paper was wetted with 1.2 mL of a 1 mM CaSO_4_ solution (control), or 1.2 mL of 20, 10, 1, and 0.1 mg L^−1^ IAA solution (Sigma, Milan, Italy) for the calibration curve, or 1.2 mL of a serial dilution of seaweed extracts. For lettuce, the experimental design was the same as for watercress except that the sterile filter paper was wetted with 1.4 mL instead of 1.2 mL, and the calibration curve was a progression of 100, 10, 1, and 0.1 mg L^−1^ GA solution (Sigma). The seeds were placed in a germination room in the dark at 25°C. After 48 h for watercress and 72 h for lettuce, seedlings were removed and the root or epicotyl lengths measured with a TESA-CAL IP67 electronic caliper (TESA, Renens, Switzerland) and Data Direct software, version 1 (ArtWare, Asti, Italy). The values obtained were the means of 20 samples and five replications, with the standard errors always 5% of the mean.

Values of hormone-like activity are reported in ppm IAA and ppm GA, expressed respectively as concentration of indoleacetic acid or gibberellic acid of equivalent activity as 1 mg C L^−1^.

### Plant material and growth conditions

Seeds of *Zea mays* L. (var. DK C6286, DeKalb, Padua Italy) were soaked in distilled water overnight and then surface-sterilized in 5% (v/v) sodium hypochlorite for 10 min, while shaking. Seeds were left to germinate for 60 h in the dark, at 25°C, on a filter paper wetted with 1 mM CaSO_4_ (Ertani et al., 2011). Germinated seedlings were transplanted into 3 L pots containing an aerated complete culture solution, with a density of 12 plants per pot. The nutrient solution was renewed every 48 h and had the following composition (μM): KH_2_PO_4_ (40), Ca(NO_3_)_2_ (200), KNO_3_ (200), MgSO_4_ (200), FeNaEDTA (10), H_3_BO_3_ (4.6), CuCl_2_ (0.036), MnCl_2_ (0.9), ZnCl_2_ (0.09), NaMoO_4_ (0.01). Plants were cultivated for 14 days inside a climatic chamber with a 14 h light/10 h dark cycle, air temperature of 27°C/21°C, relative humidity of 70/85%, and photon flux density of 280 mol m^−2^ s^−1^. Twelve days after transplant, part of plants were divided in groups and treated for 48 h with 0.5 mL L^−1^ single seaweed extracts. The remaining plants were not supplied with seaweed extracts and served as controls. Plants were randomly harvested from three pots per treatment, carefully washed and dried with blotting paper. A sub-sample of plant material was immediately frozen with liquid nitrogen and kept at −80°C for further analyses of esterase activity. Another sub-sample of plant material was dried in the oven at 65°C and used for element and sugar quantification.

### Root characteristics

Root scanning was performed before the sampling process using an Epson Expression 10000XL 1.0 system (Regent Instruments Company, Canada) as published in Ding et al. (2014). The parameters were recorded with a root image analysis system using the software WinRHIZO: main root length (mm), surface area (cm^2^), average diameter (mm), number of tips, and length of fine roots (cm) (0<L<0.5).

### Elemental composition and soluble sugars determination in maize leaves

Quantification of elements in leaves was obtained after acid digestion by using a microwave (Milestone Ethos model 1600, Milestone, Shelton, CT). Analytical-grade reagents provided by Merck (Merck, Darmstadt, Germany) were used to prepare all solutions. Water was purified using a Milli-Q system (18.2 MΩ cm, Millipore, Bedford, MA). The digestions were carried out as described in Ertani et al. (2011) inside closed Teflon vessels of 120 mL volume using approximately 500 mg dry leaf material and 10 mL of 30% (v/v) HCl. After digestion, the resulting solution was transferred and diluted with 10 mL ultrapure water. Elements were measured via Inductively Coupled Plasma Atomic Emission Spectroscopy (Spectrum CirosCCD, Kleve, Germany).

For analysis of reducing sugars, samples of leaf material from individual plants were dried for 48 h at 80°C, ground in liquid nitrogen and then 100 mg of each were extracted with 2.5 mL 0.1 N H_2_SO_4_. Samples were incubated in a heating block for 40 min at 60°C and then centrifuged at 6,000 g for 10 min at 4°C. After filtration (0.2 μm, Membra-Fil®, Whatman, Milan, Italy), the supernatants were analyzed by HPLC coupled to the refractive index detector (RI) (Perkin Elmer 410, Perkin Elmer, Norwalk, CT, USA). The soluble sugars were separated through an Aminex 87 C column (300 × 7.8 mm, BioRad, Segrate, Milan, Italy) using H_2_O as eluent at a flow rate of 0.6 mL min^−1^.

### Esterase enzyme activity

Esterase activity was performed according to Junge and Klees (1984). Leaves and roots (1 g) were homogenized (1:10, w:v) in liquid N_2_ with 0.1 M potassium acetate (pH 4.0) containing 0.1 M phosphate buffer (pH 7.0). The extracts were centrifuged at 15,000 g for 15 min at 2°C and the supernatants used as the enzyme source and expressed as a percentage of the control (0.28 OD min^−1^ fresh wt mg^−1^).

### Statistical analysis

Data represent the means of measurements on tissue material derived from plants grown in three different pots per treatment. For each analysis, five plants per treatment were used (± std). Analysis of variance (ANOVA) was followed by pair-wise *post-hoc* analyses (Student-Newman-Keuls test) to determine which means differed significantly at *p* < 0.05. To identify the structure of the interdependences of the main parameters studied, joint principal components analysis (PCA) was performed on the following 16 variables: main root length, root surface area, root average diameter, root number of tips, root length of fine roots, leaves elemental composition as calcium, magnesium, sulfur, iron, copper, manganese, molybdenum, zinc, boron, and leaves and roots esterase activity and 46 studied objects. The standardized variables were subjected to PCA; and the rotated orthogonal components (varimax rotation method) were extracted and the relative scores were determined. Only PCs with an eigenvalue >1 were considered for discussion. All statistics were made by SPSS software version 19 (SPSS inc., 1999).

## Results

### Chemical characterization and hormone-like activity of extracts

The main chemical features of the seaweed extracts assayed in this study are listed in Tables 1, **2**. Carbon (C) content ranged from 3.7 to 12.2% (g/100 mL), while nitrogen (N) content varied from 0.1 to 7.2% (g/100 mL) (Table 1). Maximum value of IPA was measured in C extract (8.45 nM), while E extract was particularly enriched in IAA (32.43 nM). D extract was the least in both hormones (2.72 and 9.70 nM for IPA and IAA, respectively).

**Table 1:** Content of total carbon (C), total nitrogen (N), IPA and IAA in extracts obtained from seaweeds *Laminaria* (A) and *Ascophyllum nodosum* spp. (B–F).

**Seaweed extract**	**C tot (%)**	**N tot (%)**	**IPA (nMol)**	**IAA (nMol)**
A	4.20	0.40	4.12	14.6
B	3.70	7.20	4.91	11.61
C	4.00	3.50	8.45	10.39
D	12.20	0.30	2.72	9.70
E	5.60	0.10	5.79	32.43
F	3.70	0.10	3.74	17.79

The amount of total phenols (TP) was extremely variable in the extracts (Table 2). In particular, *A. nodosum*–derived extracts were more enriched in phenols, especially E (1,589.4 mg L^−1^) and F (1,933.8 mg L^−1^), compared to the extract from *Laminaria* (A, 211.5 mg L^−1^). Among phenolic acids (gallic acid, protocatechuic acid, vanillic acid, caffeic acid, *p*-coumaric acid, syringic acid, *p*-hydroxybenzoic), *p*-hydrozybenzoic acid was dominant in all extracts, with values between 22.72 mg L^−1^ (E) and 81.11 mg L^−1^ (F). This phenolic compound was the unique determined in C extract. Syringic acid was exclusively identified in F, while *p*-coumaric acid was present in both A and B extracts. Gallic acid was measured in all samples except in C, and its content was maximum in E extract (33.16 mg L^−1^). A and E extracts also contained significant levels of protocatechuic, vanillic and caffeic acids.

**Table 2:** Content of total phenols and individual phenolic acids in extracts obtained from the seaweeds *Laminaria* (A) and *Ascophyllum nodosum* spp. (B–F).

**Seaweed extract**	**Tot. phenols**	**Gallic**	**Protocatechuic**	**Vanillic**	**Caffeic**	**p-Coumaric**	**Syringic**	**p-Hydroxybenzoic**
				***mg L^−1^***				
A	211.5	3.47	11.36	5.38	11.42	12.36	n.d.	38.81
B	555.9	6.19	13.25	15.24	n.d.	21.75	n.d.	50.28
C	900.4	n.d.	n.d.	n.d.	n.d.	n.d.	n.d.	58.36
D	1,244.9	4.97	n.d.	18.45	n.d.	n.d.	n.d.	50.15
E	1,589.4	33.16	16.11	8.16	5.99	n.d.	n.d.	22.72
F	1,933.8	2.90	1.97	n.d.	3.14	n.d.	4.94	81.11

The hormone-like activity of the extracts was evaluated via Audus test (Table 3). Only A and B extracts displayed GA-like activity (1.09E-07 ppm IAA and 1.34E-06 ppm GA). However, the IAA-like activity in these extracts was not detectable (B) or negligible (A). C extract was the highest in IAA-like activity (0.23 IAA ppm), followed by extracts F (0.11 IAA ppm), E (0.10 IAA ppm), and D (0.06 IAA ppm).

**Table 3:** Hormone-like activity of extracts obtained from the seaweeds *Laminaria* (A) and *Ascophyllum nodosum* spp. (B–F).

**Seaweed extract**	**IAA-like activity**	**GA-like activity**
	(ppm IAA)	(ppm GA)
A	0.01	1.09E-07
B	–	1.34E-06
C	0.23	–
D	0.06	–
E	0.10	–
F	0.11	–

*IAA- and GA-like activities are expressed as ppm IAA and ppm GA, which correspond to the concentration of either indoleacetic acid or gibberellic acid of equivalent activity as 1 mg C L^−1^*.

### Spectroscopic characterization of seaweed extracts via FT-IR and FT-Raman

**Figure 1:**
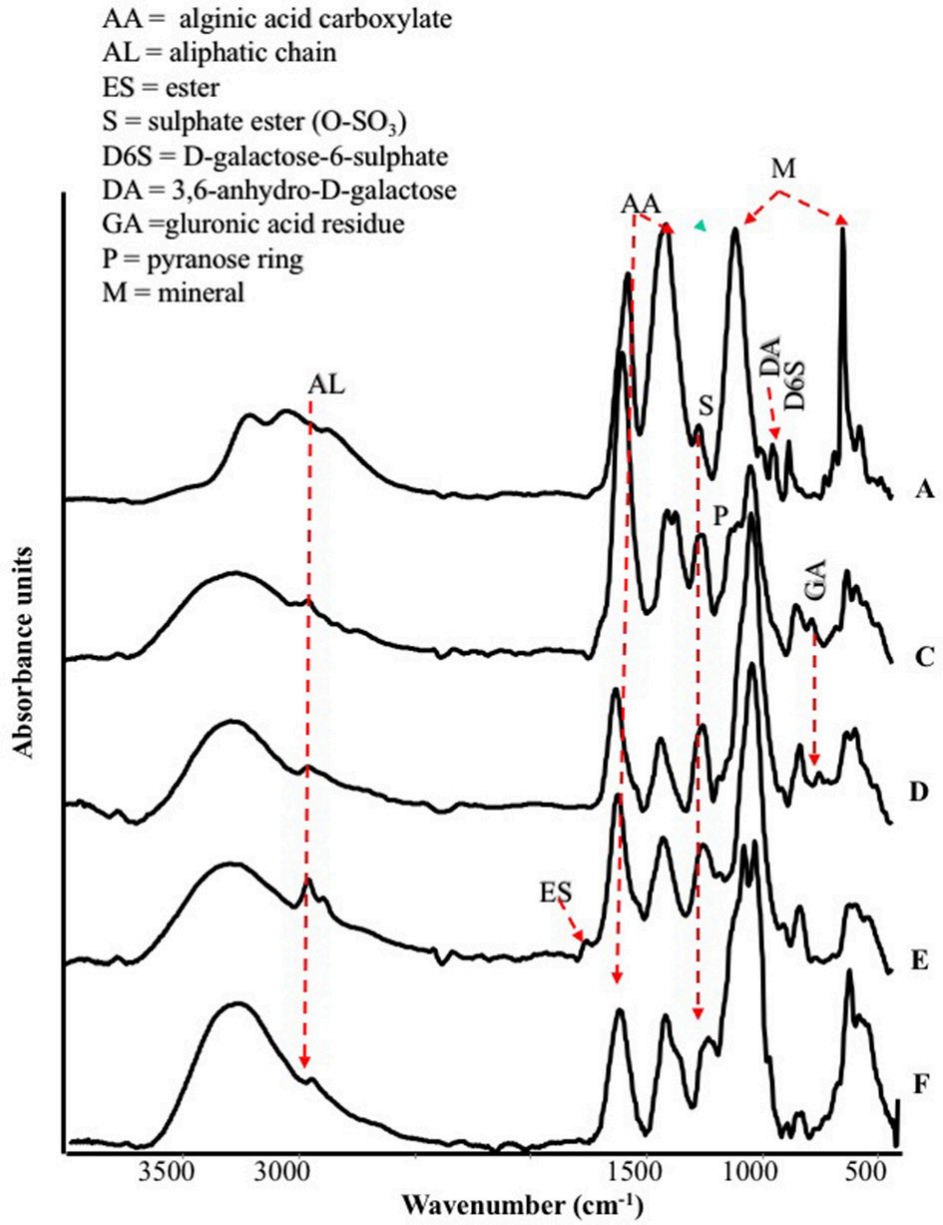
ATR/FT-IR spectra of extracts derived from *Laminaria* (A) and from *Ascophyllum nodosum* spp. (C–F).

The FT-IR spectra of different seaweed extracts are shown in Figure 1. Overall, the region included within 2,500–4,000 cm^−1^ is not particularly discriminant, except for A extract, because it displayed very similar features: a strong –OH at about 3,400 cm^−1^ and weak –CH stretching bands at about 2,950 cm^−1^. In particular, A extract differed from the other extracts because the –CH stretching bands were not observed; moreover, three components accounting for the mineral part of this sample (see below) were observed in the region 3,500–2,500 cm^−1^. The bands detected in the region between 1,800 and 600 cm^−1^ in particular, were the most characteristics for each extract, therefore they deserved a detailed description. The spectra of all extracts revealed a strong band at 1,612–1,547 cm^−1^ likely due to the asymmetric stretching of carboxylate vibration in alginic acid (Gómez-Ordó-ez and Rupérez, 2011), while the strong band within 1,412–1,380 cm^−1^ could be ascribed to the symmetric stretching vibration of the same carboxylate groups (Mathlouthi and Koenig, 1986), with the contribution of –CH and C-OH deformation vibration. The extracts also exhibited a broad band around 1,230 cm^−1^ corresponding to sulfate ester groups (S = O), which is characteristic of functional groups observed in fucoidans (Gómez-Ordó-ez and Rupérez, 2011). The bands around 1,081–1,026 cm^−1^ could be assigned to C–C–H and O–C–H deformation, C–O stretching, and C–O and C–C stretching vibrations of pyranose rings, with the contribution of mineral compounds, especially in the spectrum of sample A, whose wavenumber lies at 1,080 cm^−1^. On the other side, the spectra of the other samples show bands at lower wavenumbers (1,025–1,030 cm^−1^) due to carbohydrates and a shoulder at 1,080 cm^−1^. The band in the anomeric region between 950 and 750 cm^−1^ was typical of carbohydrates (Pereira et al., 2009, 2013). In particular, the band at 960 cm^−1^, well visible in A and F extracts, could be assigned to the C–O stretching vibration of uronic acid residues, the one observed at 814 cm^−1^, evident with different intensity in all samples, likely corresponded to C1–H deformation vibration of mannuronic acid residues, while the one recorded at 732 cm^−1^ (D and F extracts, in particular) was characteristic of glucuronic acid residues. Going into more details, E extract was also characterized by a high content in lipids, as revealed by two sets of strong C-H vibration at 2,930 cm^−1^ and 2,854 cm^−1^, which are the most intense in the spectra depicted in Figure 1, and the stretching vibration of carboxylic ester groups, evidenced by the band pinpointed at 1,734 cm^−1^ (Leal et al., 2008). Finally, the shoulder at 1,510 cm^−1^ was typical of aromatic rings vibration, consistently with the highest content of gallic and protocatechuic acids determined in E extract and of p-hydroxybenzoic acid in F extract (Table 2).

**Figure 2:**
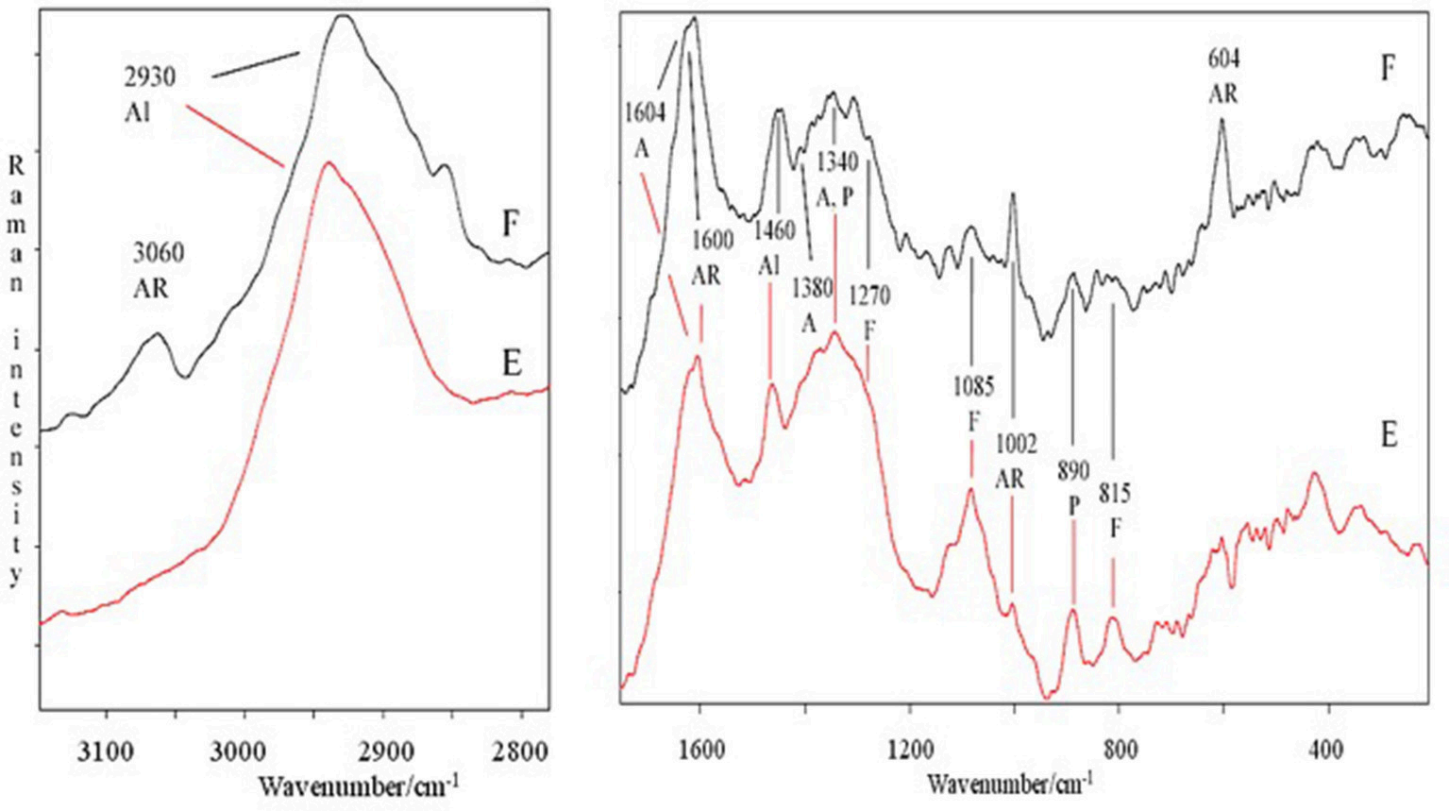
Comparison of FT-Raman spectra of E and F extracts derived from *Ascophyllum nodosum* in the 3,100–2,800 and 1,700–200 cm^−1^ regions. Abbreviations as in Figure 1; in addition, F, fucoidans.

The spectral features of extracts were also supported by FT-Raman spectra. In particular, in Figure 2 are shown, as an example, the FT-Raman spectra of two extracts (E and F). FT-Raman spectra confirmed the presence of carboxylate vibration in alginic acid at 1,604 and 1,380–1,390 cm^−1^ (shoulder) (Gómez-Ordó-ez and Rupérez, 2011). Others bands at about 2,930 and 1,460 cm^−1^ are due to the stretching and bending vibrations of aliphatic groups. These last bands were common in E and F extracts, while only in F the contemporaneous presence of the bands at 3,060, about 1,600 and 1,002 cm^−1^ indicated a moderate/high presence of aromatic groups, due to polysubstituted aromatic compounds, as the phenolic acids reported in Table 2. The bands at 1,002 cm^−1^ (the most intense normally observed in the spectra of aromatic compounds) and about 1,600 cm^−1^ are present also in the spectrum of E extract, indicating a lower presence of total aromatics/polyaromatics, as compared to F extract (Table 2). The E extract exhibited also very intense bands at 1,085 and at about 1,270 cm^−1^ (shoulder) (Synytsya et al., 2014; Marinval et al., 2016) which are typical of symmetric and asymmetric stretching of sulfate groups in fucoidans, respectively, together with the band at about 815 cm^−1^, attributable to bending of primary C6-O-S (Campos-Vallette et al., 2010; Synytsya et al., 2014). These three bands were present also in the other spectra, but with a lower intensity. Raman bands, common to both spectra, at 1,340–1,346 cm^−1^ were assigned to in-plane CCH, COH and OCH deformations in pyranoid rings with contribution of CH_2_ wagging and CH_3_ symmetric bending of galactose and fucose, respectively (Synytsya et al., 2014). Finally, the band at about 890 cm^−1^ can be generically attributed to β-glycosidic bond (Marinval et al., 2016) or to β-D-terminal in mannose or glucose containing carbohydrates (Yang and Zhang, 2009).

### Root growth in response to seaweed extracts

**Figure 3:**
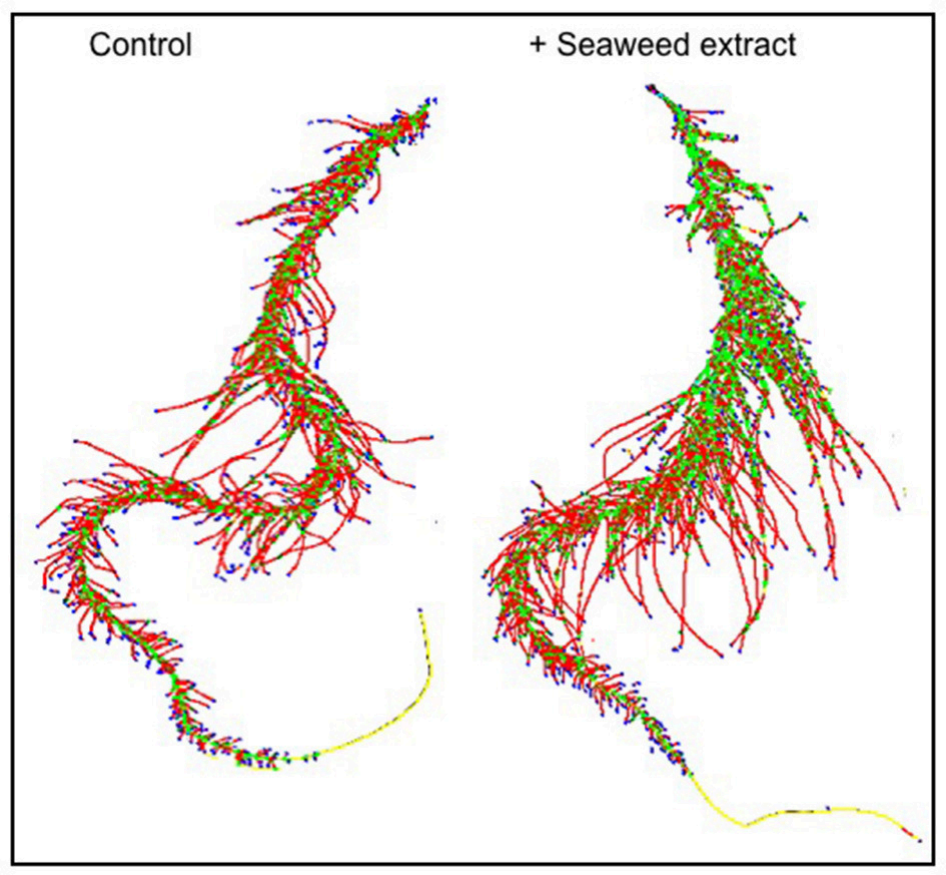
Image comparison between roots of untreated maize plants (control) and plants treated with a seaweed extract. Image was obtained using an Epson Expression 10000XL 1.0 system (Regent Instruments Company, Canada), equipped with a root image analysis system using the software WinRHIZO.

In general, root size and architecture of maize plants was positively influenced by the addition of seaweed extracts from *Laminaria* and *A. nodosum* spp. (Table 4). E extract was the most successful in stimulating root elongation (plus 81%), despite values were not significantly different from those measured for plants treated with B, C, and D extracts. Conversely, A and F extracts were the least efficient in promoting root development. In general, root surface was greater in plants supplied with seaweed extracts than in untreated plants, with maximum values measured in plants added either with C or E extract. Furthermore, plants treated with seaweed extracts, produced a higher number of root tips (plus 58 and 88% for B and E, respectively) and showed a more pronounced length of thin roots (about plus 80% for both C and E) than the controls. An example of image comparison between roots of untreated plants (control) and plants treated with a seaweed extract is depicted in Figure 3.

**Table 4:** Root growth-associated parameters of control maize plants (untreated) and plants supplied for 48 h with extracts obtained from the seaweeds *Laminaria* (A) and *Ascophyllum nodosum* (B–F).

**Treatment**	**Root length (cm)**	**Surface (cm^2^)**	**Diameter (mm)**	**Tip number**	**Fine root length (cm)**
Control	884 ± 151c	103 ± 20d	0.37 ± 0.02b	1642 ± 272d	696 ± 129d
A	1017 ± 64c	121 ± 7cd	0.38 ± 0.01b	2204 ± 197a	835 ± 58c
B	1432 ± 123ab	151 ± 13bc	0.34 ± 0.01c	2603 ± 142ab	1183 ± 110ab
C	1542 ± 68a	175 ± 8ab	0.36 ± 0.01b	2318 ± 86bc	1245 ± 57a
D	1422 ± 133ab	155 ± 18bc	0.34 ± 0.02c	2473 ± 228ab	1167 ± 107ab
E	1602 ± 140a	208 ± 21a	0.41 ± 0.01a	3092 ± 297a	1266 ± 102a
F	1115 ± 112bc	115 ± 13cd	0.32 ± 0.01c	1845 ± 238c	944 ± 88b

*Data represent the means of five measurements per treatment (± std). Different letters along the same column indicate significant differences between treatments (p < 0.05) according to Student-Newman-Keuls test*.

### Effect of seaweed extracts on the content of soluble sugars (glucose and fructose) in plants

The leaf glucose content showed great variation in response to seaweed extract application to maize plants, as shown in Table 5. Precisely, plants accumulated more glucose when treated with A, B, C, or F extracts, while a reduction in content of this sugar was evident in plants supplied with D or E (minus 38–40%). In roots, the level of glucose consistently decreased in plants treated with extracts derived from *A. nodosum*. Conversely, it did not change in plants supplied with A extract. A different trend in fructose content was observed, being the accumulation of this sugar generally higher in both leaves and roots of plants treated with the seaweed extracts (Table 5). Only plants treated with C extract accumulated less fructose than control plants (minus 38 and 24% in leaves and roots, respectively).

**Table 5:** Glucose and fructose content in leaves and roots of control maize plants (untreated) and plants supplied for 48 h with extracts obtained from the seaweeds *Laminaria* (A) and *Ascophyllum nodosum* spp. (B–F).

**Treatment**	**Glucose**		**Fructose**	
	***mg g^−1^ dwt***.			
	**Leaves**	**Roots**	**Leaves**	**Roots**
Control	9.8 ± 0.5c	29.5 ± 1.1ab	7.8 ± 1.0d	9.7 ± 1.2bc
A	12.2 ± 0.8b	32.1 ± 1.3a	11.3 ± 0.8c	9.2 ± 1.1bc
B	11.0 ± 0.4b	24.5 ± 1.5b	13.5 ± 0.5b	10.9 ± 0.4b
C	16.1 ± 0.8a	27.7 ± 1.6b	4.8 ± 0.5e	7.4 ± 1.0c
D	5.9 ± 1.3d	16.7 ± 1.3d	12.6 ± 0.7bc	10.8 ± 0.7b
E	6.1 ± 0.8d	26.5 ± 1.6b	10.7 ± 0.8c	11.8 ± 0.7ab
F	14.3 ± 1.3a	21.6 ± 1.8c	17.0 ± 0.4a	13.2 ± 1.1a

*Data represent the means of five measurements per treatment (± std). Different letters along the same column indicate significant differences between treatments (p < 0.05) according to Student-Newman-Keuls test*.

### Elemental composition in maize leaves

A number of elements (Ca, S, Mg, Fe, Cu, Mn, Mo, Zn, B) have been quantified in leaves of plants treated or not with the seaweed extracts (Table 6). Generally, the concentration of Ca, Mg, S, and Mo increased in leaves after application of extracts to plants. In particular, Ca concentration was enhanced by 3 fold in plants treated with D and F extracts, while Mg, S, and Mo concentration increased twice in plants supplied with F extract. For the remaining elements analyzed, higher accumulation was observed in leaves of plants treated with certain seaweed extracts. Boron, as an example, was more accumulated in leaves of maize only after addition of E and F extracts.

**Table 6:** Leaf elemental composition of control maize plants (untreated) and plants supplied for 48 h with extracts obtained from the seaweeds *Laminaria* (A) and *Ascophyllum nodosum* spp. (B–F).

**Treatment**	**Ca**	**Mg**	**S**	**Fe**	**Cu**	**Mn**	**Mo**	**Zn**	**B**
					***mg g^−1^ dwt***.				
Control	8.25 ± 0.12e	5.29 ± 0.09d	3.59 ± 0.22de	0.191 ± 0.005c	0.0332 ± 0.007c	0.0369 ± 0.002b	0.0017 ± 0.0001c	0.0794 ± 0.0059c	0.0198 ± 0.015b
A	13.97 ± 0.22d	7.73 ± 0.18c	3.34 ± 0.12e	0.190 ± 0.005c	0.0281 ± 0.010c	0.0343 ± 0.001b	0.0026 ± 0.0001b	0.1410 ± 0.022ab	0.0129 ± 0.010c
B	18.94 ± 0.49c	8.48 ± 0.41b	4.44 ± 0.21c	0.264 ± 0.016b	0.0302 ± 0.015c	0.0471 ± 0.004a	0.0025 ± 0.0001b	0.1604 ± 0.017a	0.0207 ± 0.007b
C	8.48 ± 0.16e	5.27 ± 0.13d	2.29 ± 0.11f	0.093 ± 0.001d	0.0439 ± 0.018a	0.0158 ± 0.001c	0.0013 ± 0.0001d	0.0778 ± 0.005c	0.0124 ± 0.012c
D	27.47 ± 0.36a	8.86 ± 0.10b	3.97 ± 0.08d	0.255 ± 0.003b	0.0475 ± 0.023a	0.0472 ± 0.001a	0.0024 ± 0.0001b	0.1040 ± 0.007bc	0.0199 ± 0.005b
E	19.70 ± 0.50c	8.75 ± 0.11b	5.32 ± 0.07b	0.275 ± 0.004b	0.0358 ± 0.005b	0.0380 ± 0.001b	0.0027 ± 0.0001b	0.0707 ± 0.001c	0.0260 ± 0.027a
F	25.72 ± 0.57b	11.07 ± 0.28a	7.51 ± 0.17a	0.330 ± 0.004a	0.0382 ± 0.008b	0.0490 ± 0.001a	0.0034 ± 0.0001a	0.0997 ± 0.004c	0.0220 ± 0.010a

*Values are expressed (mg g^−1^ d.wt.) for macroelements and Fe, while for microelements in ppm (mg kg^−1^ d.wt.). Data represent the means of five measurements per treatment (± std). Different letters along the same column indicate significant differences between treatments (p < 0.05) according to Student-Newman-Keuls test*.

Plants added with C extract did not shown any remarkable increase in nutrient content with the exception of Cu, while F extract was the most efficient in promoting plant nutrition.

### Effects of extracts on esterase enzyme activity

**Figure 4:**
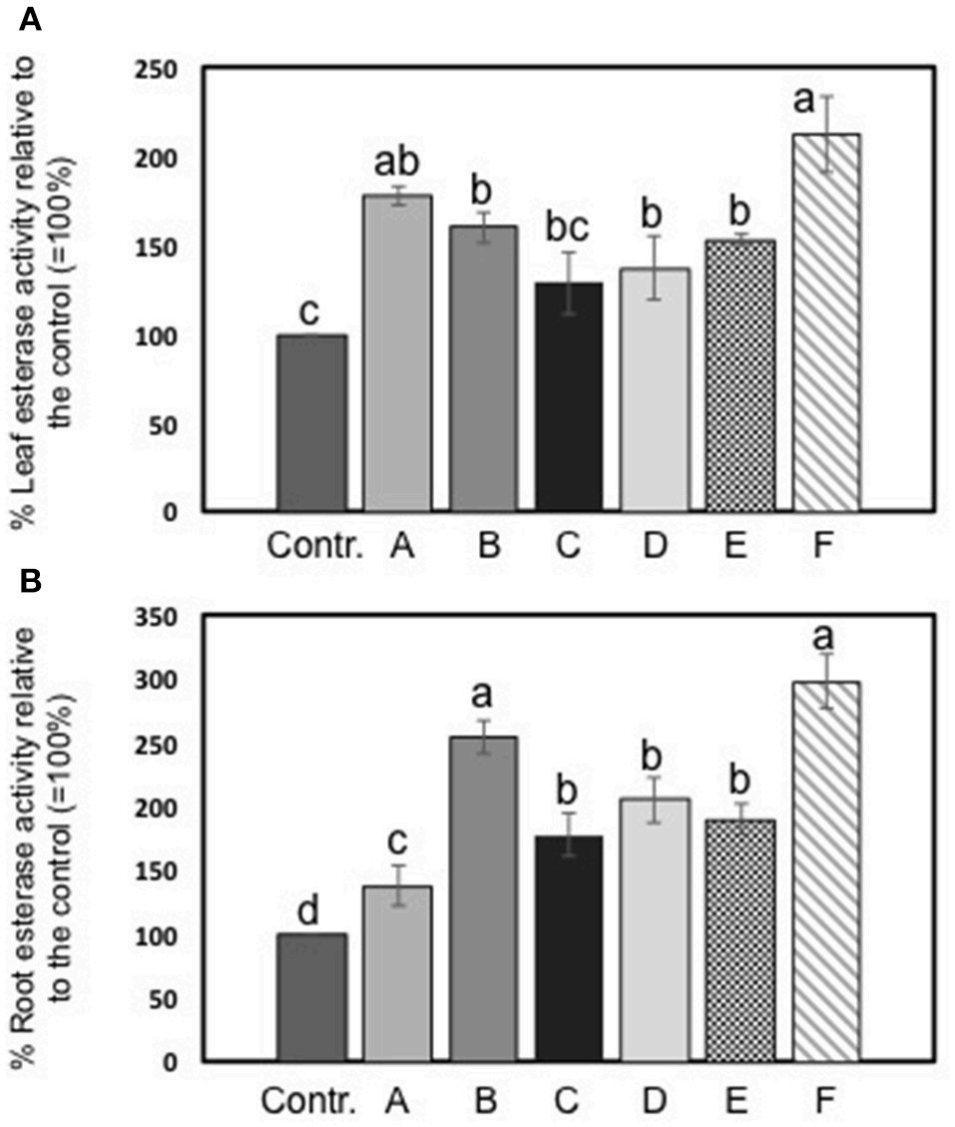
Esterase activity in leaves **(A)** and roots **(B)** of maize untreated plants and plants added for 2 days with seaweed extracts from *Laminaria* spp. (A) and *Ascophyllum nodosum* spp. (B–F). Values are expressed as percent of the control (untreated plants). Data represent the means of five measurements per treatment (± std). Different letters above bars indicate significant differences between treatments (*p* < 0.05) according to Student-Newman-Keuls test.

The activity of esterase in leaves and roots of maize plants was increased by seaweed extracts (Figures 4A,B). In particular, *Laminaria*'s extract (A) determined a more pronounced increment of esterase activity in foliar tissues than in roots (plus 38 and 78%, respectively). Among extracts derived from *A. nodosum*, F extract appeared the most effective in promoting esterase activity (plus 112% than the controls). In roots, extracts from *A. nodosum* stimulated esterase activity more than *Laminaria*'s extract. Maximum values of percent increases in activity were measured in plants treated with B (plus 154%) and F (plus 198%) extracts.

### Principal components analysis

**Figure 5:**
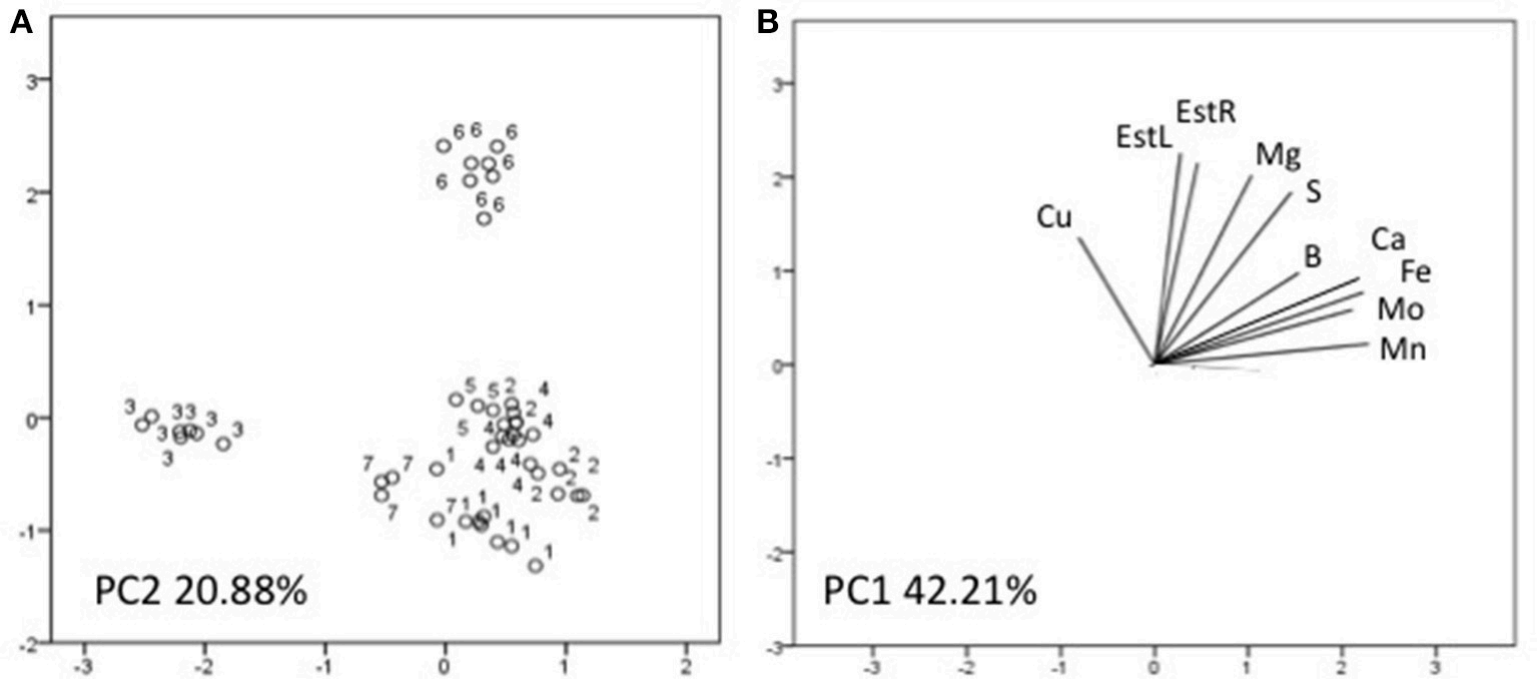
Position of the samples representative of maize plants untreated and treated with seaweed extracts **(A)** and of the variables **(B)** projected in the plane determined by the first two principal axes of the PCA. Seaweed extracts correspond to the circle number A = 1, B = 2, C = 3, D = 4, E = 5, F = 6, and untreated plants = 7. EstL and EstR: esterase activity in leaves and in roots, respectively.

**Figure 6:**
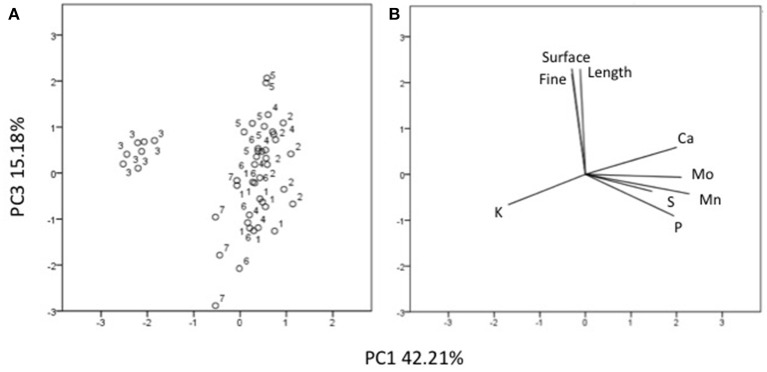
Position of the samples representative of maize plants untreated and treated with seaweed extracts **(A)** and of the variables **(B)** projected in the plane determined by the axis 1 with axis 3 of the PCA. Seaweed extracts correspond to the circle number A = 1, B = 2, C = 3, D = 4, E = 5, F = 6, and untreated plants = 7. Length = root length, surface = root surface area, fine = root fine length.

Three factors accounted for 78.27% of the total variance. Factor 1 (PC1) explained 42.21% of the variance and was positively correlated with Mn, Fe, Mo, Ca, B, Zn (Table 7). Factor 2 explained 20.88% and was positively correlated with the esterase activity in leaves and roots and the concentration of Mg, Cu and S (Table 7). Factor 3 explained the remaining 15.18% and was positively correlated with root parameters such as root surface, root length, fine roots, number of tips, and diameter (Table 7). Plotting data according to PC1 and PC2 (Figures 5A,B) allowed three clusters to be identified corresponding to maize plant treated with C extract (=3 in Figures 5, 6) and with seaweed F extract (=6 in Figures 5, 6) (top), while control plants (=7 in Figures 5, 6) and plants treated with extracts A, B, D, E (=1, 2, 4, 5, respectively, in Figures 5, 6) scattered around the origin. In particular, plants treated with F extract (=6 in the Figures 5, 6) were characterized by high values of esterase activity and Mg, whereas plants treated with C extract (=3 in Figures 5, 6) had low values in Fe, Mn, Mo, and Ca. From the projection of PC1 with PC3 (Figures 6A,B) it can be noted that untreated plants (=7 in Figures 5, 6) and plants treated with extracts A, B, D, E, F (=1, 2, 4, 5, 6, respectively, in Figures 5, 6) where distributed along a line, evidencing decreasing values in root surface, root length and fine roots from the upper (seaweed extracts B and E, = 2 and 5, respectively, in Figures 5, 6) to lower (extract A and untreated, =1 and 7, respectively, in Figures 5, 6) samples of the cluster.

**Table 7:** Loadings values of some morphometric and chemical variables on the axes identified by principal components analysis for control (untreated) maize plants and plants supplied for 48 h with extracts obtained from the seaweeds *Laminaria* (A) and *Ascophyllum nodosum* spp. (B–F).

**Variable**	**PC1**	**PC2**	**PC3**
Mn	0.940	0.091	−0.175
Fe	0.916	0.317	−0.101
Mo	0.868	0.240	−0.027
Ca	0.826	0.373	0.244
B	0.634	0.403	0.150
Zn	0.550	−0.099	−0.087
EstL	0.115	0.928	−0.195
EstR	0.190	0.885	−0.169
Mg	0.427	0.832	0.054
S	0.600	0.756	−0.153
Cu	−0.334	0.558	0.343
Surface	−0.122	−0.152	0.948
Length	−0.046	−0.063	0.946
Fine	−0.122	0.091	0.903
Tips	0.153	−0.154	0.820
Diameter	−0.076	−0.449	0.520

## Discussion

Extracts from seaweeds are rich in several bioactive compounds that might act in plants inducing an array of positive physiological responses, such as improved biomass production, amelioration of nutrition and resistance to stress (Engel et al., 2006). For this reason, they can be employed in agricultural practices to promote the health status of crops.

In this research, extracts from *Laminaria* and *A. nodosum* spp. were tested in maize plants to assay their biostimulant properties. Overall, they enhanced root system development and plant nutrition. The increased root growth by extracts is well evidenced by the PCA analysis, which highlighted a gradient where the untreated plants displayed the lowest values. Though, extracts revealed distinct chemical properties, which could explain their different efficacy in eliciting physiological responses related to plant growth and increased capacity to absorb nutrients (Figure S1). Differences in chemical composition of extracts could be ascribed to the algal species and, within the species *A. nodosum*, to different locations where the seaweeds were collected. Also, the amount of biologically active extracted compounds could vary depending on the sampling season and environmental conditions. Specifically, we found several functional groups attributable to polysaccharides (i.e., alginate or fucoidan), for which a number of biological activities have been recognized, including the stimulation of natural defense responses in plants (Hernández-Herrera et al., 2016). Spectroscopic analyses also revealed the presence of functional groups corresponding to lipids and phenols in seaweed extracts. Each type of biomolecule displayed a characteristic signal in FT-IR and Raman spectra. Even though Raman spectroscopy is an emerging tool for the study of bio-macromolecules, in our study only E and F extracts showed an acceptable signal to noise ratio, while for other extracts a strong fluorescence covered completely the weak Raman signals. Diagnostic bands of polysaccharides, mainly agars and carrageenans, were well identified by using FT-IR.

Differences in chemical composition between two *A. nodosum* commercial biostimulants, especially in terms of phenolic compounds, were reported by Goñi et al. (2016) as well. In this case, heterogeneity was evidenced between these biostimulants based on their impacts on the *Arabidopsis thaliana* transcriptome.

All extracts exerted a positive effect on the activity of esterase enzyme, which is considered a marker of plant developmental processes, being involved in organogenesis and functioning as an early indicator of somatic embryogenesis (Chibbar et al., 1988; Pedersen and Andersen, 1993; Krsnik-Rasol et al., 1999; Balen et al., 2003). Esterase belongs to a group of enzymes that hydrolyse ester bonds, and occurs in several isoforms in plant and animal cells. The increase of esterase enzymes associated with wall fractions, for instance, has been proposed to be involved in the turnover of phenolic acids that are cross-linked to wall polysaccharides (Thaker et al., 1986). Therefore, higher esterase activity in maize plants treated with *Laminaria* and *A. nodosum* spp. extracts was indicative of their stimulatory effect on plant biomass production.

Among extracts from *A. nodosum*, E was the most effective in promoting root associated parameters (root length, surface, tip number, and fine roots), as confirmed by the PCA analysis (Figure 6) as well. This was likely because of its higher content in hormones such as auxin (IAA = 32.43 nM) and cytokinin (IPA = 5.79 nM), and intermediate values of IAA-like activity. In this respect, several studies have shown that auxins and/or substances endowed with auxin-like activity contained in seaweed extracts and other biostimulants can induce positive effects on lateral root and hair formation (Mugnai et al., 2008; Pereira et al., 2009; Schiavon et al., 2010; Spinelli et al., 2010; Ertani et al., 2013a,b).

Stimulation of root development by seaweed extracts was likely responsible of increased accumulation of several macro- and micro-elements in plants, with a more pronounced effect associated to extracts derived from *A. nodosum* compared to *Laminaria*, like suggested by PCA analysis (Figures 6, **7**). The increase of minerals' accumulation in plants treated with algal extracts was previously reported and explained as a result of the up-regulation of genes coding for nitrate, sulfate, and iron transporters, and/or stimulation of root cell division and lateral root/hair development (Mugnai et al., 2008; Pereira et al., 2009; Spinelli et al., 2010).

In our study, F extract was the most successful in enhancing the plant capacity to absorb and accumulate nutrients. This extract displayed similar intermediate values of IAA-like activity as E extract, but was not efficient in improving all root parameters. We hypothesize that the remarkable effect caused by F extract on plant nutrition was primarily due to enhanced transcription and/or activity of plant membrane nutrient transporters rather than to an increase in root absorption surface. It is noteworthy that F extract was the highest in phenol content (1,933 mg L^−1^). Phenols are important signaling molecules and in adequate amounts exert several positive effects in plants, even when they are exogenously applied or present in biostimulant formulations (Ertani et al., 2011). However, if their concentration is too high in the plant or in the surrounding environment, phenol compounds might become toxic to some extent, thus overcoming the positive effects on root growth normally exerted by substances endowed with auxin-like activity (Muscolo et al., 2013). On the other hand, extracts that are very low in total phenol content may be unable to significantly increase root development as well, regardless if they display IAA-like activity. This may be the case of A extract from *Laminaria*, which was the lowest in total phenols, while displaying GA-like activity (Nardi et al., 2000).

B and D extracts determined equivalent increases in root growth, despite they greatly varied in chemical composition, especially with respect to N content, type of hormone-like activity and phenol content. Perhaps, their similar contents in IPA and IAA could have overcome differences in the type of hormone-like activity. It is also possible that nitrogen in B extract was not all promptly available for plant absorption whether in the form of long peptides, despite some of them may function as signaling molecules (Ertani et al., 2013a,b), or other organic N compounds. B and D extracts also caused a similar accumulation of numerous of elements, with the exception of Ca, Cu (for which D extract promoted higher accumulation) and Zn (more accumulated in plants treated with E), thus suggesting that active molecules in B and D extracts acted on both mineral nutrient transporters and growth signaling pathways, as it can be hypothesized for E extract.

C extract induced similar increases in root growth as B and D extracts, likely by virtue of its high content in IPA (8.45 nM) and IAA-like activity. Nevertheless, it was significantly different from all other extracts, as revealed from PCA analysis (Figures 5, 6): despite the elevate IAA-like activity and its capacity to stimulate root development, apparently it did not exert any appreciable effect on plant nutrition. Even when the total content of individual nutrients in plants treated with extract C was calculated, values were lower compared to those in plants treated with the other extracts (data not shown). Therefore, while for F extract we hypothesized that its composition was very effective in promoting plant nutrition but not root development, in this case it is possible that the formulation of C extract was more successful in eliciting responses related to root growth.

C extract also caused a decrease of fructose content in plants. Fructose seems to be related to secondary metabolites production, in particular of erythrose-4-P, which acts as a substrate for lignin and phenolic compounds synthesis (Caretto et al., 2015). The other extracts determined a general increase of fructose content, especially in leaves, which may suggest their capacity to trigger the phenlylpropanoid pathway. This metabolic route has been established as one of the main target of biostimulant action in plants (Schiavon et al., 2010; Colla et al., 2015; Ertani et al., 2017) and is critical for plant development and stress conditions overcoming. On the other side, glucose content was found to increase in leaves of plants treated with the majority of extracts, while it was reduced or unaffected in roots. Glucose in plants either acts as a substrate for cellular respiration or osmolyte to maintain cell homeostasis (Rosa et al., 2009). The reduction of this sugar, especially in the roots, may be ascribed to higher consumption because of the respiration process in order to produce more ATP for active nutrient transport.

## Conclusions

This study underlines a close relationship between the chemical properties of commercial seaweed extracts from *Laminaria* and *A. nodosum* spp. and select plant physiological responses they are able to positively induce. The results obtained strongly support the utility a robust chemical characterization of commercial seaweed extracts based on different approaches in predicting the metabolic targets of biostimulants in plants and could be extended to other seaweed biostimulants available in the market.

## Author contributions

AE analyzed the chemical characteristics of seaweed extracts and plants, and wrote part of the manuscript, OF and AT performed the spectroscopic analyses and wrote the relative part in the manuscript, DP performed the statistical analysis, root measurements and esterase activity assay, MS wrote part of the manuscript and edited it, SN supervisioned the study.

### Conflict of interest statement

The authors declare that the research was conducted in the absence of any commercial or financial relationships that could be construed as a potential conflict of interest.

## Supplementary material

Figure S1 Heat map of plant-associated parameters influenced by individual seaweed extracts. Different colors indicate different levels of induction (+, ++, +++), repression (−) or no effect (=).
