# Supplementary figures and images for: Evaluation of Seaweed Extracts From Laminaria and Ascophyllum nodosum spp. as Biostimulants in Zea mays L. Using a Combination of Chemical, Biochemical and Morphological Approaches
Source: Front Plant Sci. 2018 Apr 6;9:428. doi: 10.3389/fpls.2018.00428 (PMC5897654; doi:10.3389/fpls.2018.00428)

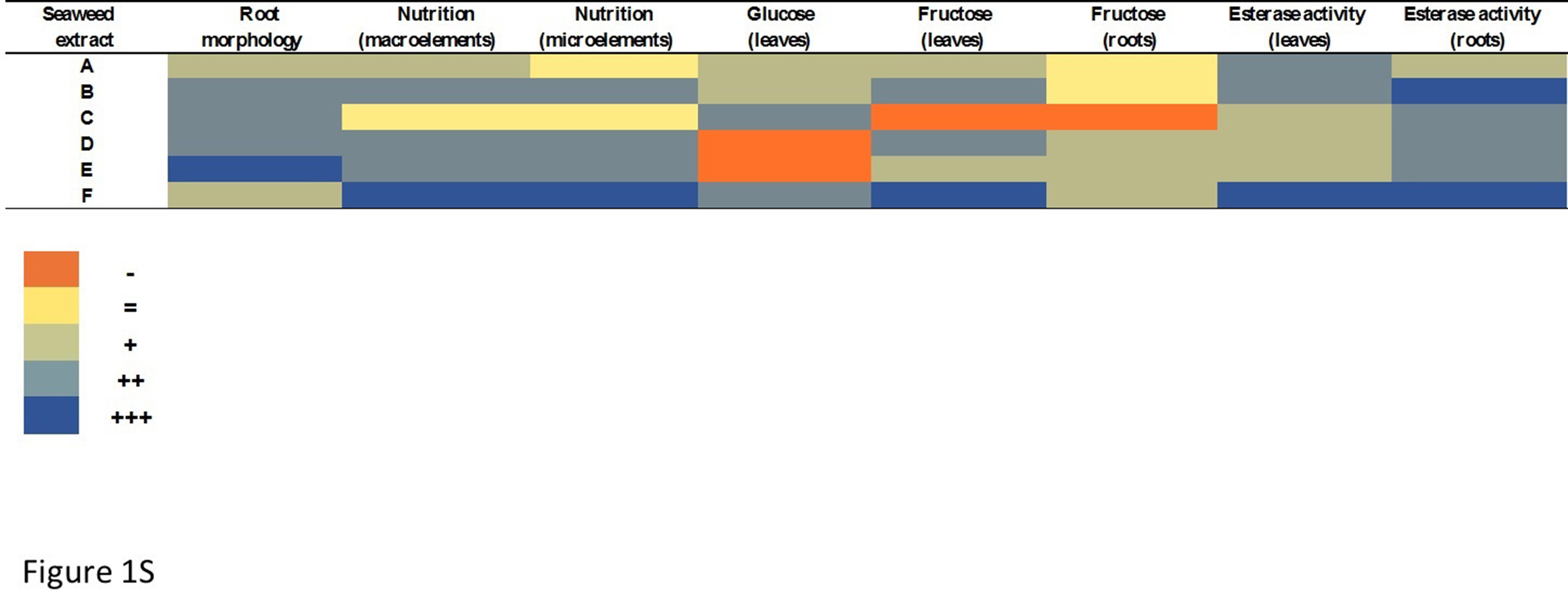

Supplement: Figure S1 — Heat map of plant-associated parameters influenced by individual seaweed extracts. Different colors indicate different levels of induction (+, ++, +++), repression (−) or no effect (=). [file Image1.TIFF]
